# Glutathione Metabolism in Plants under Stress: Beyond Reactive Oxygen Species Detoxification

**DOI:** 10.3390/metabo11090641

**Published:** 2021-09-19

**Authors:** Sonia Dorion, Jasmine C. Ouellet, Jean Rivoal

**Affiliations:** IRBV, Université de Montréal, 4101 rue Sherbrooke est, Montréal, QC H1X 2B2, Canada; sonia.dorion@umontreal.ca (S.D.); jasmine.ouellet@umontreal.ca (J.C.O.)

**Keywords:** plant stress, glutathione, oxidative stress, glyoxalase pathway, *S*-glutathionylation, reactive oxygen species, methylglyoxal, metabolic adaptation

## Abstract

Glutathione is an essential metabolite for plant life best known for its role in the control of reactive oxygen species (ROS). Glutathione is also involved in the detoxification of methylglyoxal (MG) which, much like ROS, is produced at low levels by aerobic metabolism under normal conditions. While several physiological processes depend on ROS and MG, a variety of stresses can dramatically increase their concentration leading to potentially deleterious effects. In this review, we examine the structure and the stress regulation of the pathways involved in glutathione synthesis and degradation. We provide a synthesis of the current knowledge on the glutathione-dependent glyoxalase pathway responsible for MG detoxification. We present recent developments on the organization of the glyoxalase pathway in which alternative splicing generate a number of isoforms targeted to various subcellular compartments. Stress regulation of enzymes involved in MG detoxification occurs at multiple levels. A growing number of studies show that oxidative stress promotes the covalent modification of proteins by glutathione. This post-translational modification is called *S*-glutathionylation. It affects the function of several target proteins and is relevant to stress adaptation. We address this regulatory function in an analysis of the enzymes and pathways targeted by *S*-glutathionylation.

## 1. Introduction

Glutathione (γ-glutamyl-cysteinyl-glycine) is a vital metabolite for plant survival. In the complete absence of glutathione caused by a mutation, *Arabidopsis* (*Arabidopsis thaliana*) dies at the embryonic stage [1]. While other mutants with a less severe reduction in glutathione levels can survive, they are inherently more sensitive to many biotic and abiotic stresses [2,3,4,5,6]. Total glutathione concentration is surprisingly high and is usually in the low mM range within most plant cells. This qualifies glutathione as the most abundant non-protein thiol found not only in plants but also in all three domains of life: Archaea, Bacteria, and Eukarya [7,8]. Glutathione can be found in a free state or bound to other molecules. Free glutathione exists in two reversible states that help preserve redox homeostasis within the cell. It occurs as a monomeric, reduced sulfhydryl form (GSH) and as a disulfide dimer following its oxidation (GSSG). Hereafter, GSH and GSSG respectively specify the reduced and disulfide (oxidized) forms. The term glutathione is used where the two forms are discussed or concerned. Under normal conditions, GSH is by far the most abundant of the two forms [9].

The metabolism of glutathione constitutes one of the most ancient defense strategies that have evolved to overcome the different chemical challenges resulting from aerobic life supported by respiration and glycolysis [8]. Consumption and production of molecular oxygen by aerobic metabolic processes like respiration and photosynthesis inexorably results in the formation of Reactive Oxygen Species (ROS), especially in stress conditions [10,11,12,13]. Together with ascorbate, glutathione is at the center of the mechanisms that control ROS levels in plants [14,15]. This role has been covered by several reviews over the last ten years [14,16,17,18,19,20,21]. However, the role of glutathione in plant responses to stress extends beyond the control of ROS. It also involves the detoxification of the potent glycating agent methylglyoxal (2-oxopropanal, hereafter abbreviated MG) which is an unavoidable toxic by-product of glycolysis [22]. Similarly to ROS [11,12], enhanced generation of MG is a general and common outcome in plants exposed to environmental constraints such as salinity, drought, cold, and heavy metals [23,24,25,26,27,28]. Stress-induced ROS and MG production has generated an evolutionary pressure to keep these potentially toxic compounds under control [8]. In the course of evolution, nature has optimized several glutathione-utilizing enzymes as efficient tools for their detoxification [8]. One example is the glyoxalase pathway responsible for GSH-dependent control of MG. The detoxification of MG by glyoxalases uses and generates GSH without net oxidation [29]. This is in sharp contrast to ROS detoxification by the glutathione/ascorbate cycle [14]. Oxidation of GSH to GSSG in the latter must be accompanied by recycling of GSSG to GSH. The glyoxalase pathway has recently received renewed interest in plants and our understanding of its function has seen some remarkable developments. In recent years, a number of studies have also documented the implication of glutathione in the redox-dependent post-translational modification (PTM) of proteins in plants. This process, called *S*-glutathionylation, is believed to play a protecting role against protein oxidation and mediates several plant stress responses. In this review, after briefly describing the origins of ROS and MG in plants, we discuss how glutathione metabolism (its synthesis and degradation) is regulated by stress. We next examine the components of the glyoxalase pathway and how GSH is utilized to control MG levels under stress. Finally, we review several examples in which *S*-glutathionylation is implicated in plant response to stress.

## 2. Origin of Reactive Oxygen Species and Methylglyoxal in Plant Cells

### 2.1. Reactive Oxygen Species

**Figure 1:**
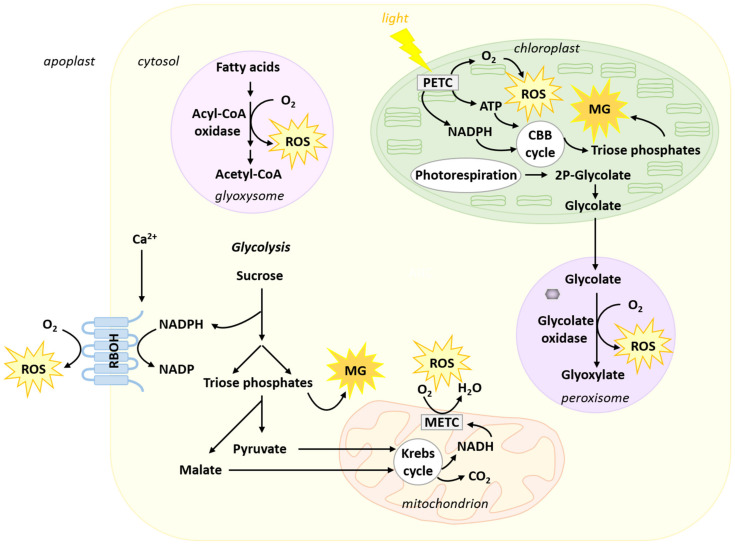
Main sites of ROS and MG formation in plant cells. ROS production occurs mainly at the levels of the mitochondrial electron transport chain (METC), the plastidial electron transport chain (PETC), the Ca^2+^-regulated Respiratory Burst Oxidase Homolog (RBOH) located at the plasma membrane, the glyoxysome and the peroxisome. MG mainly arises from triose phosphates generated during glycolysis and photosynthetic CO_2_ fixation. Further details are discussed in the text. CBB cycle: Calvin–Benson–Bassham cycle.

Plant life is inextricably linked to oxygen (Figure 1). Plants are almost exclusively photosynthetic organisms that produce oxygen through the oxidation of water by Photosystem II (PSII) [30]. Respiration also reduces oxygen to water at the level of cytochrome c oxidase and alternative oxidase in the internal mitochondrial membrane [31,32]. Electron transport chains and redox reactions involved in or associated with respiration and photosynthesis participate in the generation of ROS as by-products [33,34,35,36,37]. Successive reduction reactions from O_2_ can lead to the production of O_2_^●−^, (superoxide radical), H_2_O_2_ (hydrogen peroxide) and ^•^OH (hydroxyl radical) [34,38]. Plant cells also possess Ca^2+^-regulated Respiratory Burst Oxidase Homologs (RBOHs) at the plasma membrane [39,40] (Figure 1). These enzymes use intracellular NADPH to reduce O_2_ to O_2_^●−^ in the apoplastic space. After dismutation, O_2_^●−^ generates the more stable H_2_O_2_. The activity of this pathway is strongly associated with plant response to pathogens and their effectors [39,40,41]. Other important sources of ROS can be found in the glyoxysome and the peroxisome (Figure 1). Glyoxysomal β-oxidation of fatty acids generates ROS at the level of Acyl-CoA oxidase [42]. This pathway is important for the mobilization of oil reserves and the establishment of seedlings during germination [43]. An important peroxisomal source of ROS finds its origin in the photorespiratory pathway (Figure 1). In the chloroplast stroma, the oxygenase activity of RUBISCO leads to the formation of 2P-glycolate, which is converted to glycolate. In the peroxisome, oxidation of glycolate by glycolate oxidase leads to the generation of glyoxylate and H_2_O_2_ (Figure 1) [44]. The generation of glyoxylate eventually serves for the mitochondrial formation of glycine.

The reactivity of ROS towards macromolecules is well documented as they have deleterious effects on DNA, proteins, polyunsaturated fatty acids and carbohydrates [34,45]. Protein thiol reactivity with ROS has deep implications for protein function. Moreover, a large number of biotic and abiotic stresses increase ROS levels in plants [13,46,47,48,49,50,51,52,53]. In order to control ROS damage, plants have evolved an array of enzymatic and non-enzymatic mechanisms [14,34,37,54]. However, ROS also play an intrinsic and vital role in the normal physiology of plants [55]. Subtle changes in ROS levels can mediate signaling functions such as responses to environmental changes [37,52,56,57] which can involve modulation of the redox state of protein thiols [58] as discussed below.

### 2.2. Methylglyoxal

Reduced forms of carbon are used as substrates to support respiration. They are oxidized to CO_2_ in the mitochondrial matrix by Krebs cycle enzymes. Carbohydrates derived from starch or sucrose undoubtedly constitute the most important group of respiratory substrates. They enter the cytosolic glycolytic pathway and are used to generate pyruvate and malate, which can, in turn, feed the Krebs cycle. However, hexose catabolism by glycolysis is also associated with the production of the dicarbonyl compound MG (Figure 1). The latter is a deleterious by-product generated at low levels from triose phosphates (dihydroxyacetone phosphate (DHAP) and glyceraldehyde-3-phosphate (GAP)), mainly through a spontaneous reaction, but also enzymatically [8,59,60,61,62]. Owing to their instability, triose phosphates are susceptible to a rapid loss of α-carbonyl protons, leading to the formation of 1,2-enediolate phosphate intermediates that further undergo a spontaneous β-elimination of their phosphate groups to form MG [60]. Triose phosphate isomerase (TPI) is an abundant glycolytic enzyme that catalyses the reversible isomerization of DHAP and GAP [63]. This enzyme has evolved a structure that stabilizes the binding of the intermediate enediolate phosphate and consequently significantly reduces the formation of this undesirable side reaction [64]. Despite this, MG is continuously produced at low levels [65] and conditions favoring active glycolytic metabolism in different tissues are also conducive to the production of MG [62]. In addition to glycolysis, triose phosphates are also produced by other pathways, such as the Calvin–Benson–Bassham cycle in photosynthesis where DHAP and GAP result from the reduction of 3-phosphoglycerate. Hence, photosynthetic carbon fixation is also associated with the production of MG in the chloroplast stroma [61,66] (Figure 1). Moreover, conditions that increase photosynthetic C fixation (e.g., high light or high CO_2_) also favour the formation of MG [66]. An *Arabidopsis* mutant lacking plastidial TPI displays increased levels of MG, presumably generated non-enzymatically from DHAP [67]. MG is a highly reactive dicarbonyl metabolite [68]. Its cytotoxic properties result from the strong electrophilic nature arising from its adjacent carbonyl groups, allowing spontaneous reactions with protein amino acid residues (e.g., arginine, lysine, and cysteine), phospholipids, and nucleic acids (e.g., guanine) to form advanced glycation end products (AGEs) that impair the functionality of their biological targets [22]. Emerging evidence now indicates that MG has also a dual role in plants, which varies in a dose-dependent manner: at elevated concentrations, MG is highly toxic, while at lower levels it acts as an important signaling molecule [62,69]. Hence, the control over MG levels is crucial to maintain a balance between its beneficial and toxic effects. The MG detoxification function is fulfilled by the evolutionarily conserved glyoxalase pathway (see Section 4).

## 3. Biosynthesis, Intracellular Distribution and Degradation of Glutathione, and Their Regulation by Stresses

### 3.1. Biosynthesis and Intracellular Distribution

**Figure 2:**
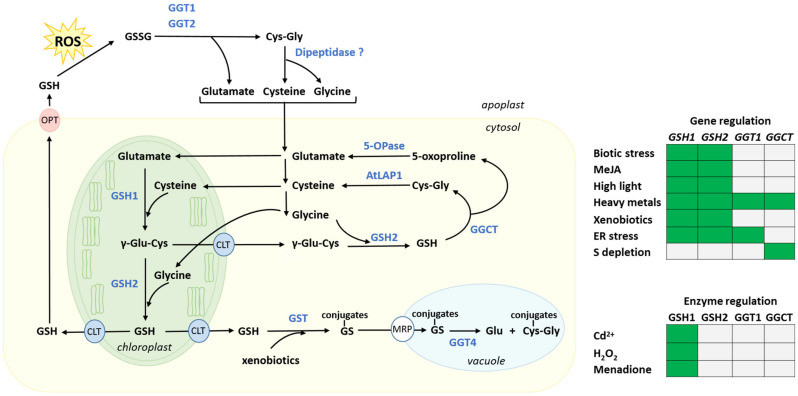
Glutathione synthesis and degradation pathways in *Arabidopsis*. Arrows indicate metabolic fluxes and inter-compartment transport. Metabolites are in black, and enzymes in blue. Known transporters are circled. A question mark indicates that experimental evidence is incomplete. GSH is synthesized in the chloroplast by the enzymes GSH1 and GSH2 from Glu, Cys and Gly. The reaction catalyzed by GSH2 also occurs in the cytosol. Degradation of GSH is catalyzed by the enzymes GGCT, AtLAP1 and 5-OPase in the cytosol. This sequence of reactions recycles the amino acids used in GSH synthesis. Degradation of GSSG occurs in the apoplast by GGT1, GGT2 and a dipeptidase still uncharacterized. This pathway also recycles Glu, Cys and Gly, which can be taken up by the cell and serve in GSH re-synthesis. GGT4 catalyzes the partial degradation of the glutathione moiety of GS conjugates in the vacuole. GSH pools are also present in mitochondria, ER and peroxisomes (not represented in the figure). The heatmap recapitulates the main regulatory features discussed in the text (regulation at the gene and enzyme levels). Green: positive effect; grey: no effect or not discussed. MeJa, methyl jasmonate.

In plants, as in other organisms, GSH is synthesized sequentially from its three amino acid building blocks by two ATP-dependent enzymes: glutamate-cysteine ligase (GSH1; EC 6.3.2.2, formerly γ-glutamylcysteine synthetase) and GSH synthase (GSH2; EC 6.3.2.3) [70] (Figure 2). The first enzyme, GSH1, conjugates Cys and Glu, generating the dipeptide γ-glutamylcysteine. GSH2 catalyzes the second step by adding Gly to γ-glutamylcysteine to form GSH. Analysis of transgenic poplars provides evidence that GSH1 is a rate-limiting enzyme for GSH synthesis [71]. In *Arabidopsis*, both enzymes are encoded by single nuclear genes [72,73,74] and are regulated by stress [75]. In this species, GSH1 is solely found in the plastids while GSH2, as a result of alternative transcription initiation sites, is dual-targeted to the cytosol and the plastids [76,77]. Furthermore, chloroplast fractionation analyses show that GSH1 and GSH2 are both found within the chloroplast stroma [77]. *Arabidopsis* knockout lines for *GSH1* or *GSH2* have lethal phenotypes at the embryo- and the early seedling-stage, respectively, underlining their essential roles for plant viability [1,78]. Complementation of the seedling-lethal *gsh2* mutant lines with cytosol-specific *GSH2* restores viability and growth. Thus, exclusive cytosolic synthesis of GSH is sufficient for normal plant growth [78].

Levels of glutathione have been quantified in various subcellular compartments in leaves by using a combination of quantitative immune-electron microscopy and HPLC analyses [79,80]. According to these studies, in absence of stress, glutathione concentrations occur in the mM range in all subcellular compartments, with the notable exceptions of the vacuole and apoplast. Highest levels are found in the mitochondria and nuclei. Since GSH synthesis is restricted to the cytosol and plastid stroma, its subcellular distribution indicates that glutathione transport likely takes place through all cellular endomembranes. Indeed, several glutathione transporters have been identified, including in the tonoplast [81,82], the mitochondrial inner membrane [83], the plastid inner envelope [84], and the plasma membrane [85,86]. At the tonoplast level, the Multidrug Resistant Proteins (MRPs) transport GSSG and different glutathione conjugates (Figure 2). At the plasma membrane, the oligopeptide transporter (OPT) mediates GSH translocation [85,86].

The use of genetically encoded redox sensitive fluorescent probes targeted to various cellular compartments shows that, under normal conditions, the glutathione redox potential (*E*_GSH_) varies considerably within the cell [87,88]. In absence of stress, the cytosolic pool of glutathione is greatly reduced with a steady-state *E*_GSH_ value around −310 to −320 mV, equivalent to maximum GSSG concentrations in the µM range and a GSH:GSSG ratio of ≈ 1000:1 to 50,000:1 [87,88,89,90]. In addition to the cytosol, mitochondria constitute a strong reducing environment, whereas the endoplasmic reticulum (ER) lumen is much more oxidizing [87]. As discussed below, the enzyme glutathione reductase (GR) is crucial for maintaining the glutathione redox potential in different compartments. This activity is absent from the ER, the vacuole and the apoplast. Therefore, in these locations, glutathione is mainly present as GSSG [16,88]. In addition, glutathione homeostasis is influenced by transport between subcellular compartments.

Exposure to stress can have strong effects on the glutathione redox status in plant cells and in different cellular compartments. For example, high light stress combined with low temperature induces a steep oxidation of the plastid glutathione pool in potato leaves [91]. Also, in response to pathogens, there is often accumulation of mitochondrial ROS which can impact the GSH:GSSG ratio in this organelle. In this context, glutathione redox status is suggested to play a regulatory role in stress responses mediated by salicylic acid [92]. These aspects of glutathione function have been extensively covered in an excellent recent review [20].

In *Arabidopsis*, export of γ-glutamylcysteine and GSH from the chloroplast to the cytosol is mediated by a family of three Chloroquine Resistance Transporter (CRT)-like transporters (CLTs) [84]. CLTs play an essential role in linking the plastid and cytosolic thiol pools to regulate GSH levels [84]. Thus, these transporters are important to generate a redox environment in the cytosol that is suitable for the fulfillment of many stress-related functions of glutathione in this compartment. These include the proper activation of mechanisms for plant defense and heavy metal detoxification. Indeed, cytosolic GSH is required for the redox regulation of Nonexpressor of Pathogenesis-Related genes 1 (NPR1) to generate its monomeric form, allowing its nuclear translocation and transcription of pathogenesis related genes [93]. GSH requirement for resistance to heavy metals is linked to its involvement in phytochelatin synthesis [94]. Accordingly, *Arabidopsis* triple mutants lacking CLTs have (i) a significant decrease in cytosolic GSH levels; (ii) are heavy metal-sensitive; (iii) have an altered NPR1-mediated systemic immunity response and (iv) are sensitive to *Phytophthora* infection [84]. Interestingly, depletion of cytosolic GSH in these mutants does not affect the decrease in leaf area induced by abiotic stresses such as high salinity or osmotic or oxidative stresses [84,95]. *OsCLT1*, a rice (*Oryza sativa*) CLT homolog, was identified by a forward genetic approach for arsenate-sensitivity [96]. Mutation of *OsCLT1* causes a decrease in cytosolic GSH levels in roots and consequently a reduced capacity for phytochelatins synthesis leading to hypersensitivity to arsenite and Cd^2+^ [96]. CLT-mediated GSH transport in rice is regulated at the transcriptional level since exposure of rice to arsenite up-regulates *OsCLT1* transcripts in roots [96]. Overall, experimental data highlight the importance of CLTs in maintaining the GSH pool and, consequently, glutathione functions.

### 3.2. Regulation of the Glutathione Synthesis Pathway

There is evidence for transcriptional regulation of the GSH biosynthetic enzymes by environmental conditions. Expression of both *GSH1* and *GSH2* is relatively ubiquitous in plant tissues but is upregulated at the transcriptional level by biotic stresses and methyl jasmonate [97], as well as abiotic stresses such as high light [9] and heavy metals [75,76], but not by H_2_O_2_ or oxidative agents [75].

Regulation of the GSH biosynthesis pathway also takes place at the post-translational level by a redox mechanism. In *Arabidopsis*, GSH1 is redox-activated by formation of an intra-molecular disulfide bridge between Cys^186^ and Cys^406^ [98]. In vitro studies show that in oxidative conditions, recombinant GSH1 forms homodimers and that reduction of the regulatory disulfide bridge causes dissociation of the homodimer into monomers [99,100]. Disruption of the dimer interface by site-directed mutagenesis recently revealed that homodimer formation of redox-activated GSH1 is not required for enzyme activation even if homodimers are expected to occur in vivo [101]. The oxidative conditions activating GSH1 (i.e., H_2_O_2_, Cd^2+^ or menadione treatment) as well as buthionine sulfoximine (an inhibitor of GSH1) [98] also increase proteolytic activity in the plastid [102,103]. It is hypothesized that GSH1 homodimerization could thus provide a greater enzyme stability through the protection of sensitive domains [101]. Since GSH1 resides in the chloroplast, the redox state of this organelle is a critical factor for the regulation of GSH synthesis. This consequently ensures prompt responses to abiotic and biotic stresses as chloroplasts are very reactive to unfavourable environmental conditions owing to the intrinsic instability of oxygenic photosynthesis [104]. It is currently unknown if the conversion from the oxidized to the reduced form of GSH1 requires a redox-coupling system. Interestingly, GSH1 is a partner of 2-Cys peroxiredoxin in co-immunoprecipitation studies [105] but the regulatory implications of this interaction remain to be determined. The redox regulation of GSH biosynthetic enzymes is instrumental in the well-established role of glutathione in the maintenance of intracellular redox homeostasis. Indeed, while metabolic conditions caused by oxidative stress create a demand for GSH and generate GSSG, these conditions are also favorable for the post-translational activation of GSH1, resulting in GSH synthesis. Consequently, increased GSH generates a reducing environment, which in turn decreases GSH1 activity and slows down GSH synthesis in a regulatory feedback loop. This regulatory mechanism allows remodelling of GSH synthesis under different stress conditions.

Immunohistochemistry and quantitative transmission electron microscopy analysis show that glutathione content in *Arabidopsis* leaves follows a diurnal cycle in several cellular compartments (i.e., mitochondria, plastids, nuclei, peroxisomes, and cytosol) [106]. Maximum glutathione amounts are present 2–3 h after exposure to light whereas minimum levels are observed at the end of the dark period. Light/dark dependent redox differences in the chloroplast stroma may also contribute to GSH1 regulation to maximize GSH synthesis simultaneously with the onset of photosynthesis [77]. Besides redox regulation of GSH1 activity, Gly and Cys availability also have a regulatory role in glutathione synthesis in plants exposed to light. In the dark, the absence of photorespiration results in Gly shortage thereby limiting GSH synthesis in leaves [107,108]. Synthesis of GSH also depends on the activity of the sulfate assimilation pathway [109]. In plants, the first organic product of S assimilation is Cys, and its availability is therefore an important determinant for GSH synthesis [71,109,110].

The glutathione biosynthetic pathway is therefore subject to multiple regulatory mechanisms. Coarse control of *GSH1* and *GSH2* expression is influenced by several environmental factors and stresses. Fine control of GSH1 activity occurs through a redox mechanism that incorporates feedback information while the activity of the pathway is also dependent on the availability of precursors.

### 3.3. Recycling of GSSG by Glutathione Reductase

In addition to the cellular concentration of glutathione, its redox status is also of prime importance in the mediation and the fine regulation of metabolism and stress responses. GSSG is effectively regenerated to GSH by the action of the flavoenzyme NADPH-dependent GR (EC 1.8.1.7) which allows repeated redox-cycling. GR participates in the maintenance of a negative *E*_GSH_ (i.e., high GSH/GSSG) to support basic processes in the cytosol, the mitochondria, and the chloroplast stroma in the light. This enzyme is essential to the function of the glutathione/ascorbate cycle [14,111]. As discussed later, GR also provides GSH needed for MG detoxification (Section 4) and for deglutathionylation of proteins by glutaredoxins (GRXs, EC 1.8.4.2) (Section 5.2.5).

#### 3.3.1. Glutathione Reductase Genes

An extensive literature review by Gill et al. [111] gathered evidence that GR activity is increased in many plant species under various abiotic stresses such as exposure to heavy metals, salinity, drought, UV radiation, and chilling temperatures. Additionally, several stress-tolerant plants display high GR activities [112,113,114,115]. Transgenic approaches to manipulate GR activity in plants also confirm that elevated GR activity plays a prominent role in improving tolerance to oxidative stress caused by a variety of abiotic factors due to efficient ROS scavenging capacity [111].

In *Arabidopsis*, GRs are encoded by two nuclear genes, *GR1* (*At3g24170*) and *GR2* (*At3g54660*), each of them coding for differentially localized isoforms. *GR1* is more expressed than *GR2* in roots whereas expression of *GR2* is more prominent than that of *GR1* in photosynthetic tissues [97]. GR1 is present in the cytosol, nucleus, and peroxisomes [116,117,118,119]. GR2 is dual-targeted to chloroplasts and mitochondria [117,120].

GRs are homodimeric enzymes [117,121]. The contribution of each isoform to total GR activity varies according to subcellular compartments and species. For example, in pea (*Pisum sativum*) leaves, GR2 activity contributes to 80% of total GR activity (77% chloroplastic and 3% mitochondrial), while GR1 constitutes the balance (20% cytosolic and ~0.4% peroxisomal) of the total activity [116,122]. The small enzymatic contribution of peroxisomal GR could be important from a signaling point of view [116]. Contrastingly in *Arabidopsis* leaves, a greater proportion (40–65%) of total GR activity is attributed to GR1 [117,123]. In an *Arabidopsis* catalase-deficient background (*cat2* mutant impaired in H_2_O_2_ removal), *GR1* is needed to respond to stress situations generating high H_2_O_2_ [123]. Despite this, *Arabidopsis* null mutants for cytosolic GR1 can grow normally even under oxidative stress conditions [117]. This absence of a distinct phenotype is explained by the presence of the cytosolic NADPH-dependent Thioredoxin Reductase (NTR)/thioredoxin (TRX) system which serves as an alternative backup system for the reduction of cytosolic GSSG, albeit with a lower efficiency than GR1 [117]. In *Arabidopsis*, this functional redundancy appears at first glance to be circumscribed to the cytosol as organellar *gr2* null mutants are lethal early in embryo development [124]. However, genetic complementation of *gr2* mutants with organelle-specific *GR2* constructs demonstrates that it is the lack of GR activity in plastids, and not in mitochondria that is responsible for embryo-lethality [125]. In the absence of mitochondrial GR2 activity, plant viability is maintained and the glutathione pool of the mitochondrial matrix is partially oxidized. GSSG does not accumulate to a toxic level because the mitochondrial NTR/TRX system can serve as an alternative for the reduction of mitochondrial GSSG [125]. In addition, the ATP-Binding Cassette (ABC) transporter ATM3 can export GSSG to the cytosol [125] where it can be reduced by GR1. Unlike mitochondria or cytosol, plastids do not contain an effective alternative system for GSSG reduction to compensate for the lack of GR activity and there is no effective GSSG export from the plastids to the cytosol. Consequently, the lack of GR2 activity in the plastid stroma results in GSSG accumulation to toxic levels that lead to early embryo lethality [125]. In the moss *Physcomitrella patens*, the loss of a mitochondrial/chloroplastic GR is not lethal, but is necessary for optimum photosynthesis under medium and high light conditions [126]. In *miao*, a plastidial *GR2 Arabidopsis* mutant displaying a moderate (50%) decrease in GR activity, the high accumulation of GSSG triggers a strong inhibition of root growth and a severe defect in root apical meristem that can be recovered by exogenous application of GSH or DTT [127]. *GR2* is thus essential for plant development under normal growth conditions, but also under stress. Indeed, antisense chloroplastic *GR2* tomato (*Solanum lycopersicum*) plants display an enhanced sensitivity to chilling stress caused by a higher accumulation of H_2_O_2_. The lower GSH regeneration in these plants results in a decrease in ascorbate redox state and ascorbate pool, and consequently a greater build-up of H_2_O_2_ [128]. Similar enhanced stress sensitivity and metabolic consequences are also observed in transgenic tobacco plants with RNAi-mediated depletion of chloroplastic GR following exposure to chilling stress [129] or methyl-viologen-induced oxidative stress [130]. In *Arabidopsis GR2* RNAi plants, elevated H_2_O_2_ levels and modified glutathione status act as signals that mediate early onset of leaf senescence dependent on age, darkness, or H_2_O_2_ treatments [124]. Exposing these plants to excess light shows that, by preventing accumulation of H_2_O_2_, GR2 activity is needed for the protection of PSII from excess light and its repair after photodamage [131].

#### 3.3.2. Regulation of Glutathione Reductases

In *Arabidopsis*, *GR2* expression is hardly affected by environmental conditions whereas weak transcriptional up-regulation of *GR1* is observed under cold stress, osmotic stress, salinity, and abscisic acid (ABA) treatment [97]. More evidence for stress regulation of *GR*s is found in other plant systems. Notably, in several graminaceous plants, *GR* gene expression is induced by Fe deficiency [132]. This induction results in increased enzyme activity. In rice, salinity stress causes a strong induction of the mRNA level of a *GR3* gene that encodes for a dually targeted (chloroplast/mitochondria) GR isoform [133]. Also, in *Hevea brasiliensis,* various stress treatments or mediators (drought, wounding, H_2_O_2_, methyl jasmonate) induce the mRNA level of a chloroplastic GR isoform [134]. To date, evidence for possible post-translational regulation mechanisms for plant GRs is still lacking.

### 3.4. Glutathione Degradation

There is abundant evidence for catabolism of glutathione in plants [135,136]. In particular, experiments with the inhibitor of GSH1 buthionine sulfoximine have helped to demonstrate the existence of an active catabolism of glutathione. Quantitative estimates in *Arabidopsis* suggest that the pool of leaf glutathione could be turning over several times per day [16]. Two independent pathways for glutathione catabolism are present in plants. One is located in the cytosol whereas the other operates in the apoplast and the vacuole (Figure 2).

#### 3.4.1. Cytosolic Glutathione Degradation and Its Regulation

The cytosolic pathway represents the main route for glutathione degradation [135]. In *Arabidopsis*, it involves three members of the ChaC/GCG sub-family of γ-glutamyl cyclotransferases (GGCTs, EC 4.3.2.9) encoded by *GGCT2;1* (*At5g26220*), *GGCT2;2* (*At4g31290*) and *GGCT2;3* (*At1g44790*). GGCTs specifically hydrolyze GSH into a cysteinyl-glycine (Cys-Gly) dipeptide and 5-oxoproline, a cyclized form of Glu [137,138] (Figure 2). These intermediary degradation products are then respectively further broken down into glutathione single amino acid constituents by (i) a member of the M17 family of metallopeptidases, the Leu aminopeptidase 1 (EC 3.4.13.18) encoded by *AtLAP1* in *Arabidopsis* [138], and (ii) by the ATP-dependent 5-oxoprolinase (5-OPase, EC 3.5.2.9) [135] (Figure 2).

The GGCT2;1 isoform has a physiological function in the response to heavy metal stress. In *Arabidopsis, GGCT2;1* mRNA levels increase upon arsenite exposure [137]. Detoxification of arsenite, thiol-reactive heavy metals, and xenobiotics requires conjugation with GSH or its oligomers phytochelatins [139,140] and consequently results in the fast depletion of cytosolic GSH. Under these conditions, GSH homeostasis is maintained by upregulation of *GSH1* and *GSH2* transcripts [75]. Additionally, increased *GGCT2;1* mRNA and GGCT activity favor adequate GSH turnover by recycling Glu, as demonstrated by isotopic labeling studies in *GGCT2;1* overexpression lines [137]. The GGCT2;1 isoform also plays a role in response to S limitation. A cis-acting SUlfur-Responsive Element (SURE) found in many genes transcriptionally activated by S depletion, is present in the promoter of *Arabidopsis GGCT2;1* [141]*. GGCT2;1* mRNA level is strongly increased (176-fold) in S-deprived *Arabidopsis* roots but the levels of the other two isoforms remain unchanged [142]. As mentioned above, S is an essential macronutrient required for the synthesis of Cys through the sulfate assimilation pathway. It has long been known that there is a close positive correlation between plant sulfate assimilation and the size of the GSH cellular pool [143,144]. The S-starvation induced *GGCT2;1* expression serves to exploit GSH as a source of Cys when S is unavailable for its *de novo* synthesis [142]. Furthermore, increased *GGCT2;1* expression under S deprivation produces a spatial redistribution of GSH in the seedling that changes root morphology to improve forage for sulfate [142].

#### 3.4.2. Plant Cytosolic Glutathione Degradation Can Be Hijacked by a Pathogen

Interestingly, a related mode of GSH degradation has been elucidated in recent years in plants infected with *Ralstonia solanacearum*, a pathogen responsible for a lethal form of bacterial wilt [145]. The pathogenic strategy of *R. solanacearum* involves the injection of more than 70 proteinic virulence factors in plant host cells. Among these, a ChaC domain-containing effector, called RipAY, was identified [145]. This protein exhibits high GGCT activity and is thought to degrade intracellular glutathione as part of a strategy to subvert plant defence systems. Moreover, RipAY requires phosphorylation of Ser^131^ to be fully active [146]. However, the protein kinase responsible for this modification has not yet been identified. A combination of in vitro and in vivo approaches demonstrated that RipAY is activated by the formation of a complex with a plant cytosolic TRX and interferes with redox signaling and suppresses plant immune responses [147,148,149]. This elegant pathogen mechanism highlights the importance of the GSH pool in plant defence.

#### 3.4.3. Apoplastic and Vacuolar Glutathione Degradation

The apoplastic and vacuolar glutathione pools are degraded by γ-glutamyl-transferases (GGTs; E.C. 2.3.2.2) which cleave the γ-glutamyl moiety of GSH, GSSG, and GS-conjugates [150,151] (Figure 2). In *Arabidopsis*, GGTs are encoded by a four genes family: *GGT1* (*At4g39640*), *GGT2* (*At4g39650*), *GGT3* (*At1g69820*), and *GGT4* (*At4g29210*). GGT1 and GGT2 isoforms are both localized in the apoplast and participate in the γ-glutamyl cycle [152]. This cycle has long been described in animal systems [153] but is still poorly-characterized in plants [154]. It involves the degradation of GSSG in the apoplast and the recycling of its amino acid constituents to resynthesize GSH in the symplast [152]. GGT1 and GGT2 degrade extracellular GSSG into Glu and Cys–Gly. The dipeptide is further broken down into Cys and Gly by a dipeptidase whose existence is inferred but not yet identified. The latter amino acids are then translocated to the cytosol for use in protein synthesis or re-synthesis of GSH followed by a novel round of export/degradation in the apoplast [155] (Figure 2). *GGT3* is considered a pseudogene. Its transcript is detected but it is unlikely to contribute to the production of a functional GGT since it is missing a sequence important for catalytic activity [156]. The *GGT4*-encoded isoform is present in the vacuole where it supports detoxification processes by initiating degradation of GSH conjugates formed by glutathione S-transferase following exposure to toxic levels of xenobiotics such as herbicides [157,158]. GSH conjugates are transported from the cytosol to the vacuole by MRPs [81,82] (Figure 2).

*GGT1* is expressed in nearly all plant tissues, although predominantly in the leaf, vascular system, and root [156,159]. For *GGT2*, expression is restricted to developing seeds, pollen, trichomes, and roots, albeit at very low levels [156,159]. *GGT4* is expressed in all tissues, but considerably more in roots [156,159]. Histochemical detection of GGT activity in different tissues of wild type *Arabidopsis* and the *ggt1* knockout mutant shows that the GGT1 isoform is responsible for more than 80% of total GGT activity in all plant tissues apart from seeds where GGT2 participates to 50% of the activity [159]. There is no visible phenotype for *ggt2* or *ggt4* null mutants [157,158,159]. The *ggt1* knockout mutant displays a shorter life cycle, a leaf-yellowing phenotype associated with higher lipid peroxidation, and build-up of GSSG in the apoplast [160]. This suggests that the major role of GGT1 is to metabolize extracellular GSSG and thereby mitigate oxidative stress [158]. The *ggt1* phenotype, with a shorter life cycle evidenced by early flowering and accelerated senescence, is interpreted as a stress response toward a general struggle in adapting to the environment [158]. Interestingly, biochemical and quantitative proteomics studies performed in leaves of wild-type *Arabidopsis* and *ggt1* null mutant show that the lack of GGT1 causes compartment-specific changes in ascorbate and GSH amounts and noticeable modifications in the levels of many proteins related to anti-oxidative and defense responses, even in the absence of environmental stimuli [161]. This remarkable constitutive reorganisation of the *ggt1* null proteome is qualified as an ‘alert response’ and is reminiscent of induced proteome rearrangements observed under different abiotic and biotic stresses [161]. This suggests that GGT1 plays a role in redox signaling from the extracellular environment to internal compartments and transmits redox information important for plant adaptation to their environment [152,161]. In connection to this proposed role, *Arabidopsis* roots exposed to Cd^2+^ display a robust and early transcriptional induction of *GGT1* during the acute stress response phase (within the first 24 h of stress) that precedes the subsequent acclimation process [162].

Recently, *GGT1* was shown to be induced by ER stress [163]. Other consequences of ER stress include induction of genes in the GSH synthesis pathway and glutathione-utilizing enzymes [163]. During ER stress, Ca^2+^ is released from ER and activates RBOHs at the plasma membrane, leading to ROS production [164,165]. In this context, it is possible that the activation of GSH synthesis helps to cope with the oxidative conditions caused by RBOHs following ER stress. Since GR is absent from the apoplast, ER stress induction of apoplastic GSSG degradation by GGT1 is a means of recycling amino acid moieties needed for GSH synthesis after oxidation of GSH in the apoplast.

From the data reviewed above, it is evident that the pathways responsible for glutathione synthesis and degradation function as integrated and interdependent units of metabolism. The metabolic structure and the built-in regulatory mechanisms controlling degradation of glutathione allow the plant cell to feed GSH synthesis and to be poised for the stress situations they may encounter.

## 4. Methylglyoxal Metabolism

The glyoxalase pathway is an evolutionarily conserved and very efficient system for the detoxification of MG that relies on GSH [29]. It takes part in the metabolite damage-control systems of organisms [166]. In plants, the glyoxalase pathway is closely linked to mechanisms implicated in adaptation to various abiotic stresses [25,27,167].

### 4.1. The Glyoxalase Pathway

**Figure 3:**
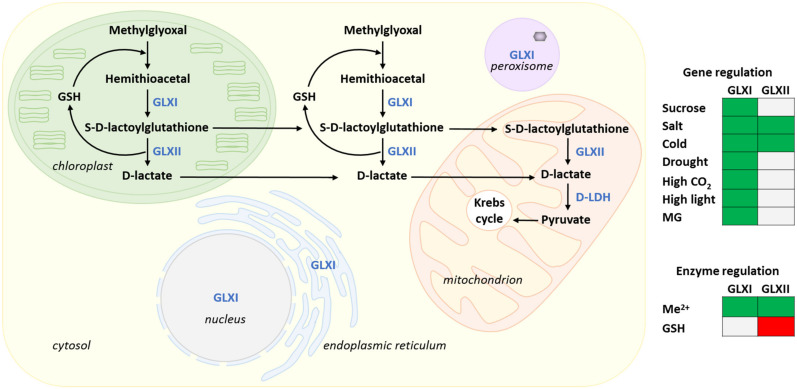
Organization and compartmentation of the glyoxalase pathway in *Arabidopsis*. Arrows indicate metabolic fluxes and inter-compartment transport. Metabolites are in black, and enzymes in blue. The enzymes GLXI and GLXII detoxify methylglyoxal to D-lactate in the cytosol and the chloroplast. The two reactions sequence uses and regenerates GSH. In the mitochondrion, D-lactate formed by GLXII is metabolized to pyruvate by a D-LDH. Isoforms of GLXI are also reported in the nucleus, the endoplasmic reticulum and the peroxisome. The heatmap recapitulates the main regulatory features discussed in the text (regulation at the gene and enzyme levels). Green: positive effect; red, negative effect; grey: no effect or not discussed. Me^2+^, requirement for divalent metal cations.

The MG detoxification process involves the consecutive action of two metalloenzymes: glyoxalase I (GLXI; S-D-lactoylglutathione lyase, EC 4.4.1.5) and glyoxalase II (GLXII; S-2-hydroxyacylglutathione hydrolase, EC 3.1.2.6) (Figure 3). GLXI isomerizes the hemithioacetal (HTA) formed by the spontaneous reaction between GSH and MG, into S-D-lactoylglutathione. The latter is hydrolyzed into non-toxic D-lactate by the action of GLXII with the release of GSH, hence effectively recycling the GSH consumed in the first step of the pathway. Using cytochrome c as an electron acceptor, mitochondrial D-lactate dehydrogenase (D-LDH, EC 1.1.2.4) catalyzes the oxidation of D-lactate to pyruvate [168,169]. Lastly, pyruvate is converted into acetyl-CoA and enters the Krebs cycle. As a final result, the cytotoxic MG is thus exploited by cells for energy production (Figure 3). In the photosynthetic prokaryote *Synechocystis* sp. strain PCC6803, one of the six glutathione-S transferases (GST Sll0067; EC 2.5.1.1.8), catalyses the conjugation of GSH with MG to form HTA, the starting point in MG detoxification by the GLX pathway [170]. This is interesting since HTA is always reported as being formed non-enzymatically from the spontaneous nucleophilic reaction between GSH and MG in both prokaryotes [171] and eukaryotes [172,173]. It is currently unknown if one member of the large higher plant *GST* gene family (e.g., 101 genes in *Glycine max*, 55 genes in *Arabidopsis* [174]) is also able to catalyze this reaction in a higher plant system. MG can also be metabolized by other enzymes that do not require GSH such as GLXIII [175,176], aldoketo reductases [177], and aldehyde dehydrogenases [178]. However, the glyoxalase pathway is considered as being the major MG detoxification system in plants [179].

### 4.2. Methylglyoxal and Stress

Increased MG levels is a general response of plant to abiotic stress [23,24,167,180]. Elevated MG production is also observed upon exposure to some biotic stresses [167,181]. In animal systems, the built-up of MG produces ROS, resulting in cellular oxidative stress [182]. In plant photosynthetic tissues, MG participates in the reduction of O_2_ and promotes oxidative stress [183,184]. Moreover, MG generates advanced glycation end-products [185] which can reduce the activities of important defense enzymes such as GSTs [186] and cytosolic ascorbate peroxidase [187] by lowering their affinity for their respective substrates. Thus, by controling the levels of MG, the GLX pathway provides an essential protection against protein glycation and oxidative stress in plants [179]. Considering its protective role, the GLX pathway has been studied in many plant species over the past 20 years, and overexpression of enzymes catalyzing the two steps, separately or together, has shown that they confer significant tolerance to several abiotic stresses such as salinity, heavy metals, and drought (reviewed in [167,188]). The tolerance observed in these transgenic plants results mainly from the decrease in MG contents. Consequently, they exhibit a reduction of oxidative damages and improved antioxidant capacity, a protection of ultra-structures in chloroplasts and mitochondria, as well as a preservation of photosynthetic efficiency under stress conditions [167].

### 4.3. Glyoxalase Pathway Enzymes

#### 4.3.1. Glyoxalases Are Encoded by Multi-Gene Families

In plants, genome wide analyses show that GLXI- and GLXII-like proteins are encoded by multi-gene families [176,189,190,191,192] as opposed to the situation observed in the majority of prokaryotes and animals, where they occur as single genes [193]. In *Arabidopsis* and other species, expression analysis of these genes reveals that several members are differentially transcribed depending on the plant tissue, stage of development, and abiotic stress [176,189,190,191,192]. However, not all genes are predicted to encode for proteins with enzyme activity. In *Arabidopsis*, amongst the eleven genes coding for GLXI-like proteins [189], only three encode isoforms with GLXI activity. According to the nomenclature of Schmitz and co-workers [194] these are (previous nomenclature [189] and gene identifiers are given in parentheses): *AtGLXI.1* (*AtGLYI6*, *At1g67280*), *AtGLXI.2* (*AtGLYI3*, *At1g11840*), and *AtGLXI.3* (*AtGLYI2*, *At1g08110*) [194,195,196]. Similarly, five *Arabidopsis* genes (*AtGLXII.1* to *AtGLXII.5*) encode GLXII-like proteins [189], but only *AtGLXII.2* (*AtGLYII5*, *At3g10850*), *AtGLXII.4* (*AtGLYII1*, *At1g06130*), and *AtGLXII.5* (*AtGLYII3*, *At2g31350*) encode for active GLXII isoforms [189,194].

#### 4.3.2. Metal Cofactors Regulation of GLXI and Product Inhibition of GLXII

According to phylogenetic analyses, AtGLXI.1 and AtGLXI.2 are classified as Ni^2+^-dependent isoforms with two GLXI domains, whereas AtGLXI.3 is a Zn^2+^-dependent one-domain isoform [195]. Previously, Ni^2+^-dependent GLXIs were believed to be strictly of prokaryotic origin, and Zn^2+^-dependent GLXIs were assumed to be of eukaryotic origin. However, it is now established that in some organisms, including *Arabidopsis*, proteins belonging to both metal activation classes are present [176,189,196,197]. Interestingly, comparative biochemical studies of recombinant GLXI isoforms using MG or glyoxal (GO) as substrates show distinct metal cofactor activation profiles [194]. GO is a reactive carbonyl species mainly arising from the degradation of glucose and glycated proteins as well as from the peroxidation of lipids [198,199]. AtGLXI.1 and AtGLXI.2 display a higher activity with Ni^2+^ using MG and GSH as substrates whereas Mn^2+^ is preferred for the conversion of GO and GSH. In contrast, AtGLXI.3 does not display this metal cofactor-dependent switch in substrate specificity and detoxifies MG and GO with high activity using Mn^2+^, Co^2+^, or Zn^2+^ as cofactors. The three isoforms use both substrates with similar affinities but display higher catalytic rates for the conversion of MG compared to GO [194]. Among the three GLXIs, AtGLXI.3 displays the highest reaction rate, with respectively 670- and 250-fold more activity than AtGLXI.1 and AtGLXI.2 [196]. It has been hypothesized that the modulation of substrate specificity by divalent cations in the GLX system may serve a yet unknown function or that other uncharacterized reactive carbonyl species could be used as potential substrates [194]. Similarly to GLXI, GLXII enzymes are also dependent of divalent cations for activity. *GLXII* enzymes belong to the metallo-β-lactamase family of proteins and contain an essential Fe^3+^/Zn^2+^ binuclear metal center [200,201,202]. In physiological conditions, total GLXI activity greatly exceeds that of GLXII [27,203]. Kinetic studies performed on the rice GLXII, OsGLYII-2, reveal that its activity can be subjected to end-product inhibition [27]. Activity of recombinant OsGLYII-2 is reduced by 50% following the addition of either 1.8 mM GSH or 280 mM D-lactate [27]. However, the relevance of inhibition by D-lactate is questionable because of the very low reported concentration of D-lactate in rice leaves (0.3 µg/g FW, i.e., ~3.3 µM) [204]. On the contrary, inhibition of GLXII activity by cellular GSH levels can be an important in vivo feature for the maintenance of a proper redox status in the cell. Indeed, stress-induced GSH consumption to form S-D-lactoylglutathione relieves inhibition of GLXII by GSH. GLXII regenerates GSH needed by GLXI until all MG is detoxified [27].

#### 4.3.3. Transgenic Manipulation of GLXs Demonstrates Their Importance for Metabolic Adaptation to Stress

There is good genetic evidence that enzymes of the GLX pathway play an important role in metabolic adaptation to stress. Heterologous expression of the three active AtGLXI isoforms in *E. coli* confers higher tolerance against various stresses (e.g., osmotic, oxidative, heat, salinity stress, and exposure to MG) and, out of the three, AtGLXI.3 contributes the most to stress tolerance [196]. In *Arabidopsis*, overexpression of these isoforms also provides improved tolerance against salinity and helps maintain lower levels of MG compared to wild type plants. Among them, plants overexpressing *AtGLXI.3* perform better than their counterparts in saline conditions [205], whereas loss of *AtGLXI.3* results in severe growth retardation [193]. Furthermore, GLXI.3 is the isoform responsible for detoxification of sugar-derived reactive carbonyl species during germination and seedling establishment as demonstrated in comparative studies of *Arabidopsis GLXI.3* loss-of-function mutant lines [194]. Hence, it appears that the highly active GLXI.3 is a main player in MG detoxification.

#### 4.3.4. Transcriptional Regulation of GLXs by Stress

In *Arabidopsis*, the transcript levels of the Ni^2+^-dependent enzyme AtGLXI.2 are increased upon sucrose feeding [194,206,207], and high CO_2_ concentrations [208]. The expression of *AtGLXI.1* and *AtGLXI.2* is also moderately induced upon stress conditions such as salt, drought, cold, and high light whereas the Zn^2+^ dependent isoform *AtGLXI.3* and its closest ortholog in rice, *OsGLYI-8*, are not responsive [189,194]. The expression of the rice *GLXII* gene, *OsGLYII-2*, is also up-regulated upon salinity stress [189]. It is suggested that the stress-dependent increase in cellular sugar levels can induce the expression of the GLX system to cope with the higher need for detoxification of sugar-derived reactive carbonyl species [194]. Indeed, cold, drought, and salt stress have an osmotic stress component that can lead to the production of soluble sugars used as osmolytes [209]. In adition, high light conditions can increase photosynthetic CO_2_ fixation, resulting in enhanced triose phosphates formation and sugar content [210,211].

Certain Ni^2+^-dependent GLXI isoforms are substrate-inducible. Indeed, *AtGLXI.2* and rice *OsGLYI-11.2* transcript levels increase upon MG exposure [189,194] through mechanisms that are still uncharacterized. Nevertheless, there are indications that MG could have signaling functions [69] and it has been shown recently that salt stress related MG accumulation results in histone methylglyoxylation affecting gene expression [212].

#### 4.3.5. Alternative Splicing of *GLX* Genes as a Mechanism Allowing Targeting of GLX Isoforms to Multiple Subcellular Compartments

The alternative splicing of *GLXI* gene products adds another level of complexity to the regulation of the GLX pathway enzymes. This post-transcriptional regulatory mechanism generates several physiologically relevant isoforms with different subcellular localisations as demonstrated using transcriptional data and expression analysis of fluorescent reporter proteins in tobacco (*Nicotiana tabacum*) leaf protoplasts [194]. *AtGLXI.1* generates a unique chloroplastic isoform. *AtGLXI.2* encodes a main cytosolic isoform and its minor splice variant localized to the ER. *GLXI.3*-encoded isoforms are present in the cytosol and the chloroplast. However, additional, and sometimes contradicting localisations for *AtGLXI.2* and *AtGLXI.3* splicing variants are also reported. For example, AtGLXI.2 is additionally reported in peroxisomes [213,214,215] and in the chloroplast [208] while AtGLXI.3 is reported in the nucleus [193]. These differences can perhaps partly be explained by the use of different experimental approaches [216]. Similarly, compartment-specific GLXII isoforms are generated by alternative splicing of *GLXII* gene products. *AtGLXII.2* generates a single cytosolic isoform, while the *AtGLXII.4* and *AtGLXII.5* splicing variants are dually targeted to the chloroplasts and mitochondria [194]. Therefore, the two GLX enzymes necessary for MG detoxification are present in both the cytosol and the chloroplast, where MG is typically generated at high levels by glycolysis and the Calvin–Benson–Bassham cycle (Figure 1 and Figure 3). These compartments are also the sites of GSH biosynthesis (Figure 2). In spinach (*Spinacia oleracea* L.), chloroplastic GLXI and GLXII activities respectively contribute to 40% and 10% of total leaf GLX activity [208]. High CO_2_ concentrations, which increase photosynthesis in C3 plants, enhance MG formation. Higher chloroplastic GLXI and GLXII activities found in these conditions are therefore related to protection against the risk of photosynthesis-linked dicarbonyl stress [208]. Since reactive carbonyl species can also be produced by several processes outside of glycolysis and photosynthesis [8,68], it is perhaps not surprising that enzymes of the GLX pathway are needed in multiple cellular compartments. The differently localized isoforms could thus provide the necessary metabolic plasticity for plant to respond to specific needs during various developmental stages or unfavourable environmental conditions [194].

## 5. Protein *S*-glutathionylation: Roles of Glutathione in the Protection and Regulation of Protein Functions under Oxidative Conditions

### 5.1. Mechanisms Involved in Protein S-glutathionylation

**Figure 4:**
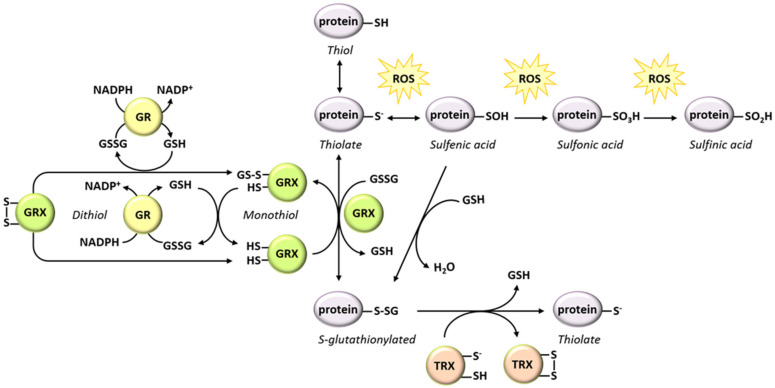
Network of reactions involved in protein oxidation, *S*-glutathionylation and deglutathionylation. The relationships between the different oxidative modifications of protein thiols and the reactions involved in protein *S*-glutathionylation and deglutathionylation are presented. *S*-glutathionylation can occur spontaneously or can be catalyzed by a GRX. Deglutathionylation is catalyzed by GRXs using a monothiol or a dithiol mechanism. It can also be carried out by a TRX, albeit at a lesser efficiency than GRXs.

As previously mentioned, various biotic and abiotic stresses lead to oxidative conditions in plant cells [217,218]. Such redox-shift often greatly affects the GSH pool which is used for scavenging stress-induced ROS [14]. In many systems, there is good evidence that stress-induced lowering of the GSH to GSSG ratio promotes the formation of mixed disulfide bridges between glutathione and protein thiols (SH groups carried by Cys residues). This reversible PTM is called *S*-glutathionylation and occurs spontaneously between glutathione and a protein thiolate (-S^−^, the deprotonated form of thiol) [217,219,220,221,222] (Figure 4). Oxidative conditions (e.g., low cellular GSH to GSSG ratio) alone are not sufficient for protein *S*-glutathionylation to occur. In a given protein, all Cys residues are not equally susceptible to this modification, as a result of the value of their p*K*_a_. A basic environment or the proximity of a metal cation are key determinants for the tendency of thiol groups to become deprotonated and consequently be affected by oxidation and spontaneous *S*-glutathionylation [222,223,224,225,226,227]. In animal systems, protein *S*-glutathionylation is also mediated enzymatically by glutathione *S*-transferase Pi [228,229,230]. Despite a recent report linking cold stress related protein *S*-glutathionylation in barley (*Hordeum vulgare*) to a higher content in glutathione *S*-transferases [231], the role of this class of enzyme in the catalysis of *S*-glutathionylation has not been formally demonstrated in plants so far. At this time, only one study provides evidence for the occurrence of catalyzed protein *S*-glutathionylation in plants [232]. In this study, *Arabidopsis* GRX C2 mediates the in vitro *S*-glutathionylation of the brassinosteroid insensitive 1-associated receptor-like kinase 1 (BAK1), leading to inhibition of its target (see Section 5.2.4). This mechanism occurs in addition to spontaneous *S*-glutathionylation favored by oxidative conditions.

### 5.2. Regulatory Effects of S-glutathionylation

*S*-glutathionylation takes place as a consequence of stress-induced oxidative conditions in plants [233,234]. Proteomic surveys reveal that various biotic and abiotic stresses induce an increase in *S*-glutathionylation of proteins implicated in metabolic processes, cytoskeleton organization, and protein turnover [233,234,235,236]. There is also evidence that protein *S*-glutathionylation happens during grain development processes in which oxidative conditions prevail [237]. In animals, *S*-glutathionylation is implicated in the protection of reactive thiolates against irreversible oxidation towards sulfinic and sulfonic acid forms [238]. In plants, this protective function is documented in vitro for the cytosolic glyceraldehyde 3-phosphate dehydrogenase (GAPDH, EC 1.2.1.12) [224] and two cytosolic NAD-dependent malate dehydrogenases (EC 1.1.1.37) under oxidative conditions [239] as well as for a chloroplastic peroxiredoxin (EC 1.11.1.27) after exposure of leaves to methyl viologen [240]. *S*-glutathionylation sometimes has no detectable effect on the target protein [241]. However, glutathione being a bulky molecule, its ligation to proteins can have an impact on their structure, function, catalytic capacity, or even subcellular localization [242,243,244]. *S*-glutathionylation of a Cys residue involved in a catalytic site can thus directly inhibit enzyme activity [224]. If the residue is located in the vicinity of the active site, its ligation to glutathione can restrict the access of the substrate to the site by causing steric hindrance [227]. Following *S*-glutathionylation, enzymes can also undergo conformational modifications, often leading to an inhibition of enzymatic activity [225,226,227]. In other cases, protein *S*-glutathionylation directly interferes with oligomerization or signal transduction [226,232].

#### 5.2.1. *S*-glutathionylation Regulates Plant Response to High Light Stress

**Figure 5:**
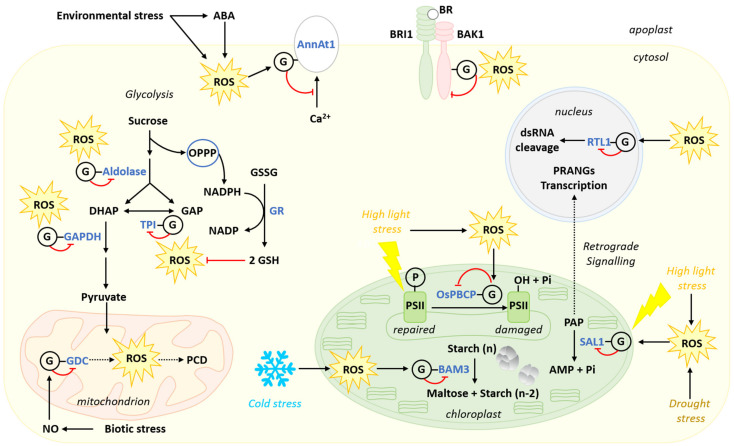
Regulation of various cellular processes by protein *S*-glutathionylation. Enzymes are in blue and metabolites in black. A circled G indicates *S*-glutathionylation of the protein. A red blunted arrow indicates an inhibitory effect. In the rice plastid, high light stress promotes the generation of ROS and the inhibition of OsPBCP by *S*-glutathionylation. The latter favors the repair of photodamaged PSII. High light stress also promotes the *S*-glutathionylation-mediated inhibition of SAL1, which is involved in the dephosphorylation of PAP. Accumulation of PAP generates a chloroplast to nucleus retrograde signal, which promotes transcription of PRANGs. Cold stress induces the *S*-glutathionylation-mediated inhibition of BAM3, which controls starch degradation in the chloroplast stroma at night. In the cytosolic glycolytic pathway, aldolase, TPI, and GAPDH are inhibited by *S*-glutathionylation under oxidative conditions, possibly favoring redirection of sucrose catabolism to the OPPP to support the production of reducing power for the conversion of GSSG to GSH. In the nucleus, ROS promote *S*-glutathionylation of RTL1 inhibiting its dsRNA cleaving activity. In the mitochondrion, biotic stress promotes *S*-glutathionylation and inhibition of GDC, which favors ROS production and PCD. At the plasma membrane of the *Arabidopsis* cell, BAK1 *S*-glutathionylation inhibits its protein kinase activity, possibly interfering with BR signal transduction. Environmental stress promotes ABA- and ROS- mediated *S*-glutathionylation of AnnAt1, decreasing its ability to bind Ca^2+^.

In plants, the redox-sensitive proteome is particularly adapted to sustain the important production of ROS by both aerobic respiration and photosynthesis [245]. In photosynthetic tissues, exposure to high levels of light or to sun flecks (increases in solar irradiance occurring in a scale of a few seconds) is a source of stress caused by high irradiation leading to oversaturation of the PETC [246]. These conditions directly and rapidly affect cellular redox balance and induce an increase in ROS with consequences at multiple levels, including rapid protein oxidation [247]. GSH levels also increase rapidly (<90 s) following high light stress to fulfill redox and signaling functions [248]. A recent survey in *Chlorella ohadii*, a green alga adapted to dry environments and extremely high light, underscores the importance of redox-dependent protein thiol modifications in response to high light conditions [236]. The proteome of this organism is enriched in Cys compared to other green algae. Sixteen percent of this proteome experiences redox changes in response to high luminosity stress. As early as 3 min after exposure to stress, most of the identified redox-responsive proteins are modified, suggesting the occurrence of redox-dependent PTMs, notably *S*-glutathionylation [236]. This increased redox-dependent regulation is potentially directly correlated to *C. ohadii* capacity to resist high light stress by efficiently modifying photosynthetic capacity to maintain growth [236]. A more direct evidence of the involvement of *S*-glutathionylation in acclimation to high light comes from rice PSII core phosphatase (OsPBCP) [227]. Under oxidative conditions induced by high luminosity, the active site of OsPBCP is *S*-glutathionylated, leading to a decrease of its activity (Figure 5). This inhibition correlates with an increase in the phosphorylation of the PSII subunits D1, D2 and CP43, necessary for disassembly and repair of damaged PSII [227]. *S*-glutathionylation of OsPBCP is thereby linked to the mechanism of redox-dependent regulation of PSII repair under high light [227].

Oxidative stress caused by high light or drought also favors *S*-glutathionylation of SAL1. SAL1 is a phosphatase catalyzing the dephosphorylation of 3′-phosphoadenosine 5′-phosphate (PAP) to AMP and inorganic phosphate (Figure 5). The modification of SAL1 by glutathione promotes the formation of an intramolecular disulfide bridge in the protein, leading to inhibition of enzyme activity. Inhibition of SAL1 results in an increase of its substrate PAP [249], which acts as a stress retrograde signal between the chloroplast and the nucleus [249,250]. Therefore, *S*-glutathionylation can indirectly influence gene regulation by affecting a signaling pathway critical for efficient response to high light stress [249]. Indeed, the accumulation of PAP can trigger the expression of the so-called Plastid Redox Associated Nuclear Genes (PRANGs) [249]. Hence, the SAL1–PAP retro-signaling pathway is important for adaptation to drought stress and response to high light in *Arabidopsis* [250].

#### 5.2.2. Stress Regulation of Carbon Metabolism by *S*-glutathionylation

Carbon metabolism enzymes are also major targets of *S-*glutathionylation in response to oxidative conditions [233,234]. In this regard, the regulation of the glycolytic pathway is particularly studied as three main glycolytic enzymes, GAPDH, aldolase, and TPI, are inhibited by *S*-glutathionylation under oxidative conditions [219,224,251,252,253]. It is proposed that inhibition of these enzymes could redirect the glycolytic carbon flux towards the oxidative pentose phosphate pathway (OPPP) to generate reductive power in the form of NADPH in plants [224,253,254,255,256] (Figure 5), as well as in other systems [257]. Besides, GRs are dependent on an adequate supply of NADPH for activity. Therefore, *S*-glutathionylation of GAPDH, aldolase and TPI can be a key element in GR-mediated glutathione homeostasis during oxidative stress (Figure 5).

The case of GAPDH is particularly well documented as *S*-glutathionylation of its catalytic Cys is demonstrated both in vitro and in vivo [224,251,258]. GAPDH activity is highly susceptible to oxidation by ROS due to the low p*K*_a_ of its catalytic Cys [224,251]. Oxidation of this residue quickly causes an irreversible inactivation of the enzyme, which can be prevented by *S*-glutathionylation [224,251]. The latter also causes inactivation of the enzyme which can be fully reversed under reductive conditions [224,251]. Nevertheless, prolonged *S*-glutathionylation of GAPDH slowly leads to the formation of insoluble globular aggregates [259]. While the latter state is irreversible, insolubilization can be partly controlled by a TRX or a GRX. Thus, control of GAPDH activity by *S*-glutathionylation under oxidative conditions needs to be finely tuned by the plant cell in order to recover enzyme activity. The length of exposure to oxidative conditions and the proportion of oxidized GAPDH probably play key roles in determining the recovery of enzyme activity following oxidative stress. The cytosolic GAPDH isoform is also a moonlighting protein [260]. Upon alteration of cellular redox, the enzyme can translocate into the nucleus or show enhanced interactions with the cytoskeleton and a mitochondrial voltage-dependent anion channel [260,261,262,263,264]. GAPDH could thus play a role in stress sensing and signaling of the redox state. However, at the present time, it is still unknown if *S*-glutathionylation of GAPDH is involved in this sensing function [256].

In *Arabidopsis* leaves, β-amylases (BAMs, EC 3.2.1.2) control a significant part of starch degradation to maltose [265] (Figure 5). The extractible activity of these enzymes is considered to be fairly stable, but different BAMs display dissimilar biochemical properties and are differently regulated by stress [265,266]. In particular, the activity of BAM3, which is primarily involved in night starch degradation in mesophyll cells, is decreased by cold stress [266,267]. Low temperatures are known to disturb cell redox status and induce oxidative stress in a variety of plant systems [48,268,269,270,271]. The cold-stress regulation of BAM3 is mainly linked to *S*-glutathionylation of the Cys^433^ residue, while two other Cys residues found near the catalytic site also undergo *S*-glutathionylation and could participate in inhibition of activity as well [267]. *BAM3* mRNA levels increase in cold stress conditions but its enzymatic activity is decreased due to *S-*glutathionylation [267]. Thus, exposure to cold temperatures results in the accumulation of BAM3 mRNA and inactive BAM3 enzyme, allowing preservation of starch during stress. It is hypothesized that this mechanism could increase plant recovery after stress, allowing quick resumption of starch degradation once cells are back to a more reduced state [267]. Although this regulatory mechanism is not characterized in vivo, it is argued that BAM3 *S*-glutathionylation allows for a differential regulation of BAM1 and BAM3 during stress. Activity of BAM1, a guard cell isoform involved in day-time starch degradation, is regulated through the formation and reduction of an intramolecular disulfide bridge [267]. The Cys residue modified by *S*-glutathionylation in BAM3 is conserved in BAM1. However, physiological conditions do not promote this modification in BAM1. *S*-glutathionylation of BAM3 thus appears to be responsible for the specific regulation of this isoform by cold stress [267].

To summarize, mounting evidence suggests that *S-*glutathionylation plays an important role in the regulation of several primary metabolism enzymes, and could impact carbon metabolism during conditions inducing oxidative stress. Increased flux through the OPPP would limit the impacts of oxidative stress by providing NADPH to GR for the recycling of GSSG to GSH used in the control of ROS. *S*-glutathionylation of BAM3 likely preserves starch during cold stress, thereby easing plant recovery after stress.

#### 5.2.3. *S*-glutathionylation in Biotic Stress

*S-*glutathionylation is also implicated in the response to biotic stress in plants. Plants infected by pathogens induce H_2_O_2_ production (oxidative burst) as part of the hypersensitive response, which is potentiated by nitric oxide (NO) [272,273,274,275,276,277]. Plant cells challenged with an increase in H_2_O_2_ use their antioxidant machinery to keep it under control [14]. In turn, this raises the concentration of GSSG [92,278,279,280] together with distinct changes in glutathione contents in different subcellular compartments [20]. There is also evidence for the implication of *S*-glutathionylation as part of a regulatory mechanism of oxidative burst. Indeed, in response to NO, mitochondrial glycine decarboxylase (GDC, EC 1.4.4.2) is inhibited by *S*-glutathionylation [281]. This inhibition leads to a decrease in mitochondrial NADH production that could lead to a partial reduction of oxygen at the level of the mitochondrial electron transport chain (METC) [281]. The exact mechanistic details involved are still uncertain, but the process triggers an oxidative burst leading to the programed cell death observed in response to bacterial infection [281]. Furthermore, *Mesembryanthemum crystallinum* infected by *Botrytis cinerea* accumulates *S*-glutathionylated proteins [280]. Another survey performed on *Arabidopsis* cells after salicylate and flagellin treatments highlights the importance of redox regulation of the proteome during the immune response [282] since more than 3000 proteins are redox-modified by a short (15 min) treatment. However, the precise regulatory mechanism in play for most of these proteins remains to be determined.

Redox regulation also appears to be an important component of the function of ribonuclease three-like (RTL) proteins involved in the control of small interfering RNAs (siRNAs) [283,284]. RTLs mediate the cleavage of double stranded RNAs and are important in response to viral infection in eukaryotes [285]. For example, the redox PTM of the siRNA suppressor RTL1 has been characterized in some details. RTL1 is subject to regulation of its activity by *S*-glutathionylation [284]. The protein is modified in vitro by GSSG on Cys^230^, a residue essential for RNAse activity, also conserved in Dicer-Like1 proteins [284]. Upon modification, RTL1 RNA-cleaving activity is inhibited. This redox-dependent control of RTL1 could play an important role in the regulation of small RNA biogenesis and accumulation of coding and non-coding RNA in response to environmental conditions [284]. In addition, RTL1 *S*-glutathionylation appears to be relevant to the cellular oxidative conditions prevailing in plant cells after viral infection [284,286].

#### 5.2.4. Implication of *S*-glutathionylation in Hormonal Regulation of Stress Response

The leucine-rich repeat receptor-like protein kinase BAK1 at the plasma membrane is known to associate with brassinosteroid-insensitive 1 (BRI1), a brassinosteroid (BR) receptor [287]. Proper expression of both *BAK1* and *BRI1*, presumably providing adequate BR signaling, is beneficial for *Arabidopsis* resistance to salt stress [288]. BAK1 also has a crucial role in pathogen-associated molecular pattern (PAMP) immunity because it interacts with Flagellin Sensing 2, the receptor of bacterial flagellin, a PAMP signal [289]. This interaction is critical for proper immune response against plant pathogenic microbes [289]. In the BAK1 cytoplasmic domain, Cys^353^, Cys^374^, and Cys^408^ can be spontaneously modified by glutathione in oxidative conditions, whereas GRXC2 more specifically mediates the *S*-glutathionylation of Cys^353^ and Cys^408^ [232]. This modulation of BAK1 activity is demonstrated in vitro, using BRI1-derived peptides as substrates for the protein kinase activity. It constitutes the first published example of a plant protein kinase regulation by *S*-glutathionylation [232] and has potential implications in BR signal transduction and plant immunity. Further protein modelization studies strongly suggest that the modification of Cys^408^ exerts a negative allosteric effect on BAK1 protein kinase activity, without eliminating the possibility that *S*-glutathionylation of the other two Cys also participate in the process [225].

Protein *S*-glutathionylation is also suggested to play a role at the interface between changes in cellular redox state and hormonal signal transduction pathways under stress conditions. In *Arabidopsis,* this PTM links the stress hormone ABA to Ca^2+^ signaling via the regulation of annexin1 (AnnAt1), a protein which has protective effect against drought stress [290]. Annexins are Ca^2+^ and membrane binding proteins and several reports also document their activity as Ca^2+^ channels [291]. Manipulation of *AnnAt1* expression demonstrates that AnnAt1 can reduce H_2_O_2_ levels *in planta*. Recombinant AnnAt1 is *S*-glutathionylated in vitro by GSSG, leading to a decrease in its binding affinity for Ca^2+^ [290]. Evidence for in vivo *S*-glutathionylation comes from heterologous expression of *AnnAt1* in *Nicotiana benthamiana* after leaf infiltration with ABA [290]. It can be hypothesized that oxidative stress or oxidative conditions induced by ABA lead to *S*-glutathionylation of AnnAt1, affecting its Ca^2+^ binding capacity. The improved resistance to drought stress is possibly related to the protective effect of AnnAt1 against H_2_O_2_ [290]. This AnnAt1 regulatory mechanism could thus represent a way to mediate crosstalk between oxidative stress caused by ABA and Ca^2+^ signaling pathways.

Ethylene mediates a number of processes in plant development and stress responses [292]. Glutathione is implicated at several levels in the regulation of ethylene biosynthesis, with subsequent impact on stress resistance improvement. Exogenous GSH induces ethylene biosynthesis by up regulating the transcript levels of enzymes responsible for ethylene production, 1-aminocyclopropane-1-carboxylate (ACC) oxidase (ACO, EC 1.14.17.4) and ACC synthase (EC 4.4.1.14) [293]. Moreover, GSH increases *ACO* mRNA stability and ACO protein level while ACO is a target for *S*-glutathionylation in vitro [293]. It is hypothesized that the latter could impact ACO protein activity or stability [293]. However, most of the evidence concerning redox regulation of ACO comes from experiments conducted in vitro and much still remains to be done to support the view that *S*-glutathionylation of ACO actually regulates ethylene signal transduction pathways and response to stress.

#### 5.2.5. Glutathione Implication in Protein Deglutathionylation

*S*-glutathionylation of proteins is spontaneously reversible in reducing conditions, but the process can be accelerated by enzymes. In plants, GRXs [224,253,284], and to a lesser extent TRXs (EC 1.8.1.9) [224], can efficiently catalyze the removal of glutathione from proteins. After reaction, TRXs are recycled by TRX reductases that depend on reducing power coming from NADPH or reduced ferredoxin. GRXs can have a monothiol or a dithiol catalytic mechanism [90]. Both mechanisms require GSH for the removal of glutathione from proteins by GRXs. Hence, an increase in the GSH to GSSG ratio favors protein deglutathionylation. In the deglutathionylation reaction, GSH becomes oxidized to GSSG, which is recycled to GSH by GR. Thus, as noted above, GR is an essential component of the deglutathionylation of proteins. Deglutathionylation of glutathionylated targets by GRXs is always associated with reactivation of target activity. This is the case for deglutathionylation of GAPDH by GRXC1 [224], TPI by GRXC1 and GRXC2 [253], and RTL1 by GRXC1 [284].

## 6. Conclusions

The aim of the present work was to review several aspects of glutathione metabolism in plants in response to stress. GSH is needed to detoxify two by-products of aerobic metabolism: ROS and MG. We focused our review on glutathione metabolism outside of its well-known function in ROS detoxification. Synthesis and degradation of glutathione are highly regulated by environmental conditions. This regulation occurs at the transcriptional and posttranslational levels, emphasizing the great flexibility that is built-up in the metabolism of this molecule to respond to biotic and abiotic environmental challenges. In addition, glutathione metabolism participates in the control of MG under stress in plants. Much like ROS, MG generates a hormesis response. Under normal conditions, an equilibrium between production and removal of ROS and MG maintains them at a basal level necessary for optimum plant growth and development. However, various stresses can perturb this equilibrium. Indeed, a common outcome in plants subjected to environmental constraint (e.g., salinity, drought, cold, heavy metals, pathogens) is an enhanced level of ROS and MG, which have deleterious effects on multiple classes of biomolecules. Glutathione is used by the glyoxalase pathway to detoxify MG. Enzymes of this pathway are important for adaptation to environmental stresses; subject to exquisitely complex transcriptional and posttranscriptional regulatory mechanisms; and highly regulated by environmental conditions. ROS and MG detoxification under stress both compete for a common pool of GSH. It would be of great interest to investigate how glutathione redox status and pool size depletion would impact these two competing processes. This area of glutathione biology appears to be understudied at the present time. Finally, we reviewed the implication of glutathione in protein PTM. Studies of *S*-glutathionylation of specific target proteins have recently produced evidence demonstrating that glutathione is implicated at another level in plant response to stress. Glutathione can thus be considered as a genuine Swiss army knife in the plant toolbox of metabolic adaptations to environmental stresses.
